# Medical Literature

**Published:** 1867-06

**Authors:** 


### MEDICAL LITERATURE.

Dr. Post, of New York, read a report on Medical Literature, in which he mentioned among other publications of the year, the Report of the New York Board of Health, in regard to Cholera.

Dr. Sayre found fault with the report in that it dignified the production of the New York Board of Health with the name of Medical Literature. He moved to strike out that portion.

The motion was not seconded, and the report was referred for publication.

The Association then adjourned until 9 o'clock to-morrow morning.

